# Labial Frenotomy for Symptomatic Isolated Upper Lip Tie

**DOI:** 10.7759/cureus.32755

**Published:** 2022-12-20

**Authors:** Cecilia G Freeman, Jason F Ohlstein, Nicholas A Rossi, John B McIntire, Luis D Neve, Shiva Daram, Harold S Pine

**Affiliations:** 1 Otolaryngology-Head and Neck Surgery, St. Luke's University Health Network, Bethlehem, USA; 2 Otolaryngology-Head and Neck Surgery, St. Luke’s University Health Network, Bethlehem, USA; 3 Otolaryngology-Head and Neck Surgery, Specialty Physician Associates, Bethlehem, USA; 4 Otolaryngology-Head and Neck Surgery, University of Texas Medical Branch, Galveston, USA; 5 Facial Plastic and Reconstructive Surgery, Premier Dermatology and Cosmetic Surgery, Newark, USA

**Keywords:** pediatric otolaryngology, frenotomy, otolaryngology, breastfeeding, labial frenotomy, pediatrics, feeding difficulties, ankyloglossia, upper lip tie

## Abstract

Background: The benefits and challenges of successful breastfeeding for both mother and child have been well-established in the literature. While ankyloglossia, or tongue tie, alone or in combination with upper lip tie has been the focus of several previous studies, very few have directly addressed isolated symptomatic upper lip tie and the role of surgical correction for breastfeeding difficulties.

Materials and methods: Seven infants with isolated upper lip tie and breastfeeding difficulty were taken to the operating room for labial frenotomy. These infants were assessed at their follow-up visits for their degree of weight gain since their procedure. Their mothers were surveyed regarding their experiences with breastfeeding since the frenotomy was performed.

Results: In this article, we present seven infants with isolated upper lip tie and breastfeeding difficulty who were treated with labial frenotomy. Subsequently, these infants demonstrated improved weight gain, and all mothers reported increased ease of breastfeeding.

Conclusion: These findings implicate lip tie as an underrecognized cause of breastfeeding difficulty and suggest that labial frenotomy is an effective treatment in these patients. Larger-scale randomized controlled studies are necessary to further evaluate this topic.

## Introduction

A thickened maxillary labial frenulum, or upper lip tie, may impede an infant’s ability to latch onto a mother’s breast, potentially resulting in feeding difficulty [1]. While the literature has demonstrated benefit from frenotomy in patients with tongue tie and patients with concurrent tongue tie and lip tie, to date, there has been no published high-level evidence to show benefit from frenotomy in patients with isolated lip tie [2,3]. In this article, we report seven infants who presented with breastfeeding difficulty and improper latching. They were ultimately diagnosed with an isolated upper lip tie and underwent surgical frenotomy with excellent results.

## Materials and methods

Seven patients with isolated lip tie, without concurrent tongue tie, and breastfeeding difficulty were identified retrospectively at the University of Texas Medical Branch Hospital over the course of a year. Patients greater than three months of age and those with concomitant tongue tie were excluded from the study. The patients ranged in age from one to 12 weeks at the time of surgery. Frenotomy was conducted in the operating room under either local anesthesia or general anesthesia via a facemask. Following anesthesia, the upper lip was manually retracted cephalad. A Colorado Needle monopolar electrocautery with a coagulation setting of 8 was then used to release the maxillary labial frenulum. This was performed until a diamond-shaped defect was evident. The patients were subsequently transferred back to the care of anesthesia without complication. Postoperative instructions for all patients included were consistent. For the first two weeks following the procedure, parents were instructed to massage the upper lip three times a day.

At follow-up, patient weight was recorded, and mothers were asked about the quality of breastfeeding. The mothers were surveyed as to whether or not they noticed a subjective improvement in breastfeeding since the procedure was performed. Patient weight was recorded both at the time of surgery and their subsequent follow-up appointment. The patient weight at presentation for surgery was subtracted from the patient weight at their follow-up visit, in order to calculate the gross weight change over that time period, as measured in kg. This number was subsequently divided by the number of weeks that passed between the patient's surgery and their follow-up visit. This yielded the mean weight change per week as measured in kg/week. This calculation was performed in order to provide a more comparable value given the variable time to follow-up among patients. Given the small sample size, no statistical analysis was performed with these values. Rather, they are presented here in order to demonstrate trends and to inspire further research into the topic. This study was exempt from the University of Texas Medical Branch (UTMB) Institutional Review Board (IRB) review.

## Results

All patients tolerated the procedure well without any surgical or anesthesia complications. At follow-up, all seven patients demonstrated weight gain since frenotomy, with a mean increase of 0.597 kg. The average weight gain per week was 0.257 kg. This value was determined, utilizing the formula described in Materials and Methods, in order to illustrate trends in the rate of weight gain observed in these infants following frenotomy. Additionally, each mother was surveyed about their experiences postoperatively with feeding their infants. All mothers in this series reported subjectively improved ease and quality of breastfeeding (Table 1).

**Table 1:** Weight Change Following Frenotomy

Case #	Age at surgery (weeks)	Time to follow-up (weeks)	Weight change (kg)	Weight change/week (kg/wk)	Feeding improved
1	3	2	0.574	0.287	Yes
2	1	4	1.27	0.3175	Yes
3	1	1	0.41	0.41	Yes
4	7	1	0.355	0.355	Yes
5	4	2	0.256	0.128	Yes
6	12	1	0.096	0.096	Yes
7	6	6	1.22	0.203	Yes

**Figure 1:**
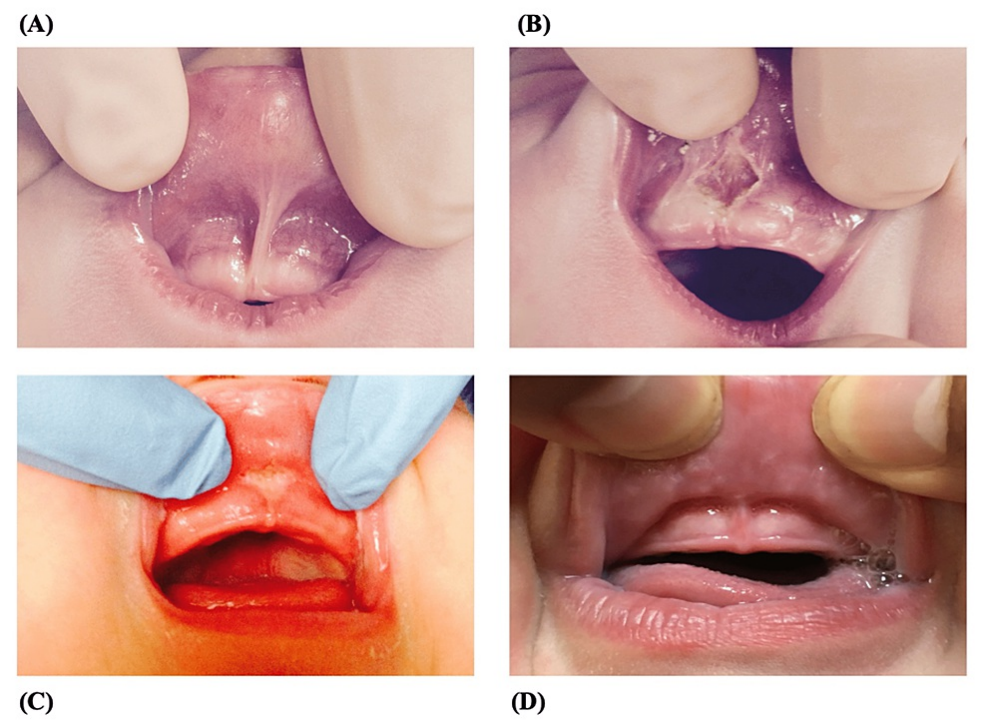
Example of Upper Lip Tie and Post-Operative Appearances Following Frenotomy (A) Pre-operative appearance of a patient with an upper lip tie. (B) Immediately following operative frenotomy; note the diamond-shaped defect. (C and D) Post-operative follow-up pictures demonstrating partial and full healing of the frenotomy site.

Four representative photos are included in Figure 1. Figure 1A depicts an example of the preoperative appearance of the maxillary labial frenulum. Figure 1B depicts the appearance of the frenulum while still in the operating room, immediately after the frenotomy was performed. Figures 1C, 1D represent the frenulum in two stages of the healing process, with Figure 1C representing an image of a partially healed frenotomy site, and Figure 1D representing the final result which has completely healed. No instances of re-tethering or regrowth of the frenulum were seen at follow-up in any of the patients included in this series.

## Discussion

The benefits of breastfeeding for infants are well-known and can include optimal nutrition, improved development, and potentially decreased rates of infections, allergies, obesity, and diabetes [4]. Further benefits of breastfeeding, as compared to breast pumping, include maternal-infant bonding, a soothing effect for both mother and baby, and improved self-esteem in mothers [5-9].

Although there are many benefits to breastfeeding, it relies on a complex and easily disturbed mechanism. Correct latch is attained when the lips freely flange outward with mucous membranes contacting maternal skin. Next, the tongue moves in a cupping and upward motion to pull the breast into the mouth toward the palate. An intraoral vacuum is generated, resulting in milk expulsion [10,11]. An infant with an oral cavity anomaly may be unable to perform this process properly. In these infants, a shallow latch may occur, during which the infant clamps down on the base or tip of the nipple. This abnormal process may cause the mother pain and discomfort with feeding, which may ultimately result in the discontinuation of breastfeeding [11,12]. Therefore, it follows that part of the assessment of infants with breastfeeding difficulty should include evaluation for oral cavity anomalies such as tongue tie or lip tie [3,9,13].

Both tongue tie and lip tie have been associated with breastfeeding difficulty [1,3,11]. Frenotomy has been shown to improve feeding and decrease maternal pain with feeding in infants with tongue tie and infants with tongue tie and lip tie. However, there is little literature to support the positive effects in the case of an isolated labial frenotomy [2,3,10-12,14,15]. Recently, Patel et al. [16] conducted a study evaluating 22 infants with isolated upper lip tie who underwent frenotomy in the office. Following frenotomy, utilizing a similar technique as we report here, they conducted a survey by phone to assess changes in the breastfeeding experience. A majority of mothers reported improvement in overall satisfaction with breastfeeding (73%), reduced nipple pain if present at baseline (73%), improved latch (82%), and reduced noises during feeding if present at baseline (67%). Additionally, 54% of mothers felt their infants received more milk [16]. To our knowledge, their study is the only published article in the literature to date that assesses breastfeeding outcomes after frenotomy for an isolated upper lip tie.

Lip tie was first described by Griffith in 1904, and over the years, it has been the subject of controversy, along with tongue tie, and it is debated whether this condition may contribute to breastfeeding difficulty [17]. With a lip tie, the newborn’s upper lip is prevented from flanging outward, which interferes with the normal infant sucking mechanism as described above [1,11]. To better characterize this anomaly on physical examination, Kotlow previously described a lip tie classification system with a grading system from 1 to 4 [11]. Another classification system, the Stanford scale, was developed in 2017 by Santa Maria et al. which is essentially a simplification of the Kotlow system, instead grading 1 to 3 in hopes of increasing inter-rater reliability [18]. In a recent study, this increase in inter-rater reliability was confirmed. This study by Shah et al. also graded the degree of upper lip tie in 100 infants using these two scales and found no correlation between the degree of upper lip tie and comfort with breastfeeding [19]. However, there remains controversy in grading the severity of upper lip tie using these scales as both scales are entirely based on the insertion point of the gingival attachment of the maxillary labial frenulum, and they do not consider other anatomic characteristics of the frenula or any functional characteristics such as flexibility [20].

Patel et al. [16] chose to utilize more functional characteristics, such as difficulty in manually everting the lip during physical examination or the mother reporting the infant’s lip frequently rolling under during feeding, as criteria for frenotomy in lieu of degree of tethering on either scale. In addition, the mothers included in their case series also reported clicking noises during feeding, seepage of milk from the corners of the mouth, and excessive air entry or gulping leading to child discomfort [16]. In contrast, the newborns included in the study performed by Shah et al. [19] were all healthy infants evaluated in the mother-baby unit and did not present to the otolaryngology office secondary to breastfeeding difficulties. The findings in these two studies, among others, suggest that the anatomical appearance of the maxillary frenulum alone cannot reliably predict breastfeeding outcomes and therefore should likely not be utilized as the sole criteria for frenotomy in the absence of any reported difficulty with breastfeeding [18-20].

The ideal treatment of symptomatic lip tie continues to be debated, with options including non-surgical, cold steel, electrocautery, and lasers [9,12,15,21-23]. The most straightforward option is frenotomy, in which the frenulum is simply divided. Due to concern that the frenulum may become re-tethered, some authors advocate for frenectomy, in which the frenulum is excised entirely [24]. Other authors recommend more complex procedures such as V-Y plasty or Z-plasty [21,24,25]. In our study, frenotomy with monopolar electrocautery was shown to be effective in improving breastfeeding, with no instances of the frenulum becoming re-tethered. It also resulted in a mean weight gain of 0.257 kg/week in our patients. Newborns from birth to age six months are expected to grow about 5-7 ounces each week [26-28]. An average weight gain of 6 oz per week is equivalent to 0.17 kg/week. Although this study does not have a large enough sample size to quantitatively compare these, it is worth noting the trend of increased average weight gain/week compared to the national mean after frenotomy, possibly suggesting an accelerated rate of weight gain following improvement in breastfeeding.

The limitations of this study include its retrospective nature and small sample size. However, as this controversial topic continues to gain more attention in the news, social media, and among professional societies, we believe this study to be an important piece of information for caregivers and to help build the foundation for larger, controlled, prospective studies in the future. This study supports the findings of Patel et al. [16] by presenting similar outcomes at another institution and adds to the sparse existing literature as only the second paper to investigate breastfeeding outcomes after frenotomy for an isolated upper lip tie. Finally, outside of the experience reported in this study, it has been the author’s anecdotal experience that nearly all infants presenting with an isolated upper lip tie, or concurrent upper lip and tongue ties, had a dramatic improvement in feeding following frenotomy.

## Conclusions

In summary, lip tie is a condition that is gaining acceptance for its association with breastfeeding difficulty. These patients present with breastfeeding difficulty and an abnormal latch, which can be simply and effectively addressed with frenotomy. Patients should be carefully selected for this procedure based on physical examination and history. While a larger study population and prospective data are needed to generate stronger conclusions and guide society's recommendations, this study demonstrates a successful improvement in breastfeeding and weight gain following the correction of isolated upper lip tie.
